# A single-institution retrospective analysis of intraoperative radiation boost during breast-conservation treatment for breast cancer

**DOI:** 10.1007/s00432-022-04534-9

**Published:** 2022-12-25

**Authors:** Franka Hochhertz, Peter Hass, Burkard Röllich, Hans-Joachim Ochel, Ahmed Gawish

**Affiliations:** 1 Department of Radiation Oncology, University Hospital Magdeburg, Leipziger Str. 44, 39120 Magdeburg, Germany; 2 Department of Radiation Oncology, Erfurt Helios Hospital, Erfurt, Germany

**Keywords:** Early breast cancer, Breast-conservation therapy, Adjuvant radiation therapy, Boost, Intraoperative radiotherapy

## Abstract

**Background:**

As part of a breast-conservation strategy for breast cancer, there are presently no data from randomized controlled studies on the use of intraoperative radiation (IORT) as a tumor bed boost. The effectiveness and safety of IORT as a boost therapy at a tertiary cancer center were retrospectively reviewed in this study.

**Methods:**

Patients had breast-conserving surgery from 2012 to 2016 that included staging of the axillary lymph nodes, a single dose of 20 Gy IORT with 50-kV photons, whole-breast irradiation (WBI), and (neo-)adjuvant systemic treatment (if applicable). During the follow-up patients were monitored for the assessment of acute and late toxicities (using the Common Terminology Criteria for Adverse Events version 4.03). Results included ipsilateral (IBTR), contralateral (CBE), and distant metastasis-free (DMFS) breast progression-free survival, as well as overall survival (OS).

**Results:**

The 68 patients had a median follow-up of 91.5 months (with a range of 9–125). Most patients (*n* = 51) had T1 disease and were clinically node negative. Only a small number of individuals had triple negative or high-grade illness. The majority of patients had sentinel node biopsy, and three (4.4%) had to have their tumors removed again since their original margins were positive. Finally, there were no distinct tumor bed margins. Neoadjuvant chemotherapy was administered to ten (14.7%). The median duration from BCS to WBI was 54.5 days, and conventionally fractionated WBI was used to accomplish WBI most frequently (*n* = 57, 96.6%). IORT was administered in a single 20 Gy dosage. 50 Gy was the median WBI dosage (range 40.05–50.4 Gy). There were no grade 4 adverse events for any patients in. Toxicities following surgery were minimal. There were only one patient with grade 3 toxicity (radiation dermatitis) to observe. Five tumor bed recurrences and two contralateral breast incident each occurred.

**Conclusion:**

This work adds to the preliminary evidence already in the literature and supports the use of IORT in boost settings. When randomized trials like TARGIT-B are eventually published, these hopeful findings should be prospectively evaluated.

## Introduction

After breast-conserving surgery (BCS), radiotherapy (RT) lowers the chance of local recurrence and death from breast cancer (Cancer and Trialists’ Collaborative G et al. 2011). When a focal RT boost is given to the tumor bed after whole-breast irradiation (WBI), the risk of local recurrence goes down even more (Bartelink et al. 2015). External-beam RT is the most common way to treat breast cancer after surgery, but other methods are becoming more and more popular. Intraoperative RT (IORT) is one type of treatment that has been used in place of WBI and as a boost (Harris and Small 2017; Pilar et al. 2017). Randomized data for the first setting come from the TARGIT-A and ELIOT trials (Vaidya et al. 2014; Veronesi et al. 2013), as well as the TARGIT-B (NCT01792726) and HIOB (NCT01343459) trials, which are still going on and just came out (Fastner et al. 2020). IORT is given as a single fraction during BCS using either electrons or 50-kV X-ray therapy. Most of the research on IORT has been done on low-risk patients. The idea that IORT could be used instead of WBI on its own was criticized. The current data show a tendency for higher local recurrence rates. Because of this, the authors suggest using IORT alone without WBI on only the most carefully chosen patients (Valente et al. 2021). Theoretically, IORT has a few benefits, including "same-day approach" settings and more convenience for the patient (Coombs et al. 2016). IORT saves more skin and prevents a possible repopulation of the tumor between the end of surgery and the start of WBI. It also makes it easier to get a better idea of the size of the operative tumor bed, which could lead to less radiation being used. Compared to the scar boost that uses electrons, there is less chance that the target will be missed because it is hard to find the tumor bed. Notably, current guidelines allow a safe and repeatable boost definition even after oncoplastic surgery (Strnad et al. 2015). Because there aren't any published data from randomized trials of IORT as a boost, institutions need to share their experiences. The goal of this retrospective study from a single institution was to describe the results and side effects of using 50-kV X-rays as an IORT boost for early breast cancer.

## Materials and methods

### Patients and treatment

Patients with breast cancer who received a single dose of 20 Gy IORT as a tumor bed boost from 2012 to 2016 were included in this research. According to institutional protocols, BCS with sentinel lymph node excision or axillary nodal dissection was carried out. Based on individual recommendations from the multidisciplinary oncological board and presently relevant guidelines, neoadjuvant or adjuvant chemotherapy as well as endocrine treatment were given.

The procedure for IORT was as follows: after tumor excision, a single IORT dose of 20 Gy was applied skin-sparingly referenced to the applicator surface (range 20–50 mm) using 50-kV X-rays generated by an intrabeam X-ray device (Carl Zeiss Surgical, Oberkochen, Germany). With this technique, the dose is attenuated down to 5 Gy at 1 cm from the edge of the excision cavity. During IORT, extra care was given to reduce skin exposure.

Following the completion of wound healing, WBI was administered using conventional fractionation (50–50.4 Gy in 25–28 fractions) or hypofractionation (40.05 Gy in 15 fractions) in accordance with established institutional standards.

Oncentra MasterPlan, (Nucletron, Veenendaal, the Netherlands) were used to do CT-based three-dimensional treatment planning. The radiotherapy (6 or 18 MV) was delivered by means of a linear accelerator (Artiste, Siemens, Erlangen, Germany). Deep-inspiration breath hold WBI was given to patients with left-sided breast cancer. Patients with estrogen receptor (ER)-positive illness underwent adjuvant endocrine treatment for 5–10 years after WBI.

For the first three years, a breast ultrasound was conducted every six months. Six months after WBI, and then once a year following the first mammogram, mammograms were taken. Biopsy results confirmed suspected recurrences. At the routine follow-up visits, an assessment of acute and late toxicity is performed using the Common Terminology Criteria for Adverse Events (CTCAE).

The primary outcome is the cumulative local recurrence rate (LR), which takes into account any ipsilateral in-breast tumor recurrence (IBTR) confirmed by imaging and/or biopsy. Secondary objectives include cumulative regional (nodal) recurrence rates (RR), contralateral breast progression-free survival (CBE), progression-free survival rates (PFS), defined as recurrence at any place other than the breast and overall survival (OS). All definitions started with the IORT date and ended with the relevant occurrence. The Kaplan–Meier technique was used to determine survival times. A mean, median (range), and frequencies are used to report data. To assess the effects of several factors (applicator size, systemic treatment, or fractionation regimen) on acute and late toxicity, binary correlation analysis using Spearman rank correlation was performed. Statistics were considered significant for P-values below 0.05. SPSS version 28 was used for the statistics (IBM, Armonk, NY, USA).

## Results

There was a total of 68 patients gathered, all of whom had IDC. The range of ages was 37.8–79.3 years, with a mean age of 55.93 years, median 54.6. The recommended IORT dosage was 20 Gy in a single fraction (fx), and the average beam-on duration was 25 min (range 24–28). 17 patients had lymph nodes that were positive on final pathology, 14 with a single axillary lymph node (N1). Three re-excisions were performed on three patients with positive surgical margins, 65/68 of the patients achieved negative surgical margins. 85% (*n* = 58) possessed positive progesterone receptors, 60 patients were estrogen positive, whereas 21% (*n* = 14) possessed positive Her-2. Only four patients had triple negative, and three patients have G3 high-grade disease Table 1 shows the patient and treatment demographics. Neoadjuvant chemotherapy was administered to 10/68 (15%) patients, while adjuvant Chemotherapy was offered in 20 patients. Hormonal therapies were administered in 64/68 patients, 51 patients received tamoxifen, only nine patients received Letrozole and only four patients underwent Herceptin-therapy.

**Table 1:** Patients characteristics

	Range	*N* = 68	Percent	Median
Age	37.8–79.3			54.65
Tumor	T1b	17	25	
	T1c	34	50	
	T2	14	21	
	T3	2	3	
	T4	1	1	
Nodal status	N0	51	75	
	N1	14	21	
	N2	2	3	
	N3	1	1	
ER+		60	88	
PR+		58	85	
Her2+		14	21	
Triple negative		4	94	
Operation	BET	65	96	
	Mastectomy	3	4	
Side	Left	32	47	
	Right	36	53	
Radiotherapy	Adjuvant	59	87	
Chemotherapy	Neoadjuvant	10	14.7	
	Adjuvant	20	29.4	
Hormontherapy	Tamoxifen, letrozol, herceptin	64	94	
Mammography in FU		56	82	
Death		9	13	

Only 59 patients underwent WBI, nine patients didn’t receive WBI. The median period from BCS to WBI was 54.5 days (range 21–325), and the great majority (*n* = 57, 82.4%) got conventionally fractionated WBI, only two patients received hypofractioned RT. the median WBI dosage was 50.4 Gy (range 40.05–51 Gy). These patients openly rejected WBI at their own desire or because they were too old, but they nonetheless received intensive clinical monitoring and adjuvant systemic treatment. The regional lymph node levels were treated with normofractionated RT using 50.4 Gy over 28 fractions for all patients with pN+ (*n* = 17).

In every case, IORT was effectively given at a median dosage of 20 Gy utilizing a median 40 mm applicator surface (range 20–50 mm). In the majority of cases (60%), WBI was implemented utilizing conventional tangential treatment portals. Only one patient received IORT with 10 Gy for a 6 mm left-sided IDC, to reduce the dose of the thorax-wall.

### Toxicity

The toxicity profile of the study population is shown in Tables 2 and 3. Grade 2 and 3 toxicities following WBI were restricted to 8 (18.2%) incidences of radiation dermatitis, while grade 4 events were not seen by any patients in acute toxicity. Prior to WBI, postoperative toxicity was minor, there are only 2 patients with grade 2 seroma. Grade 1 hematoma/ erythema were reported in 9 cases. Additionally, there was just 1 instance of a late grade 3 of radiation dermatitis as adverse event.

**Table 2:** Acute toxicity < 90 days after IORT, based on the CTCAE criteria

Adverse event	Grade 0 (%)	Grade 1 (%)	Grade 2 (%)	Grade 3 (%)	Grade 4 (%)	*N* = 44 (%)
Hyperpigmentation	95.4	2.3	2.3	0	0	100
Skin pain	84.1	13.6	2.3	0	0	100
Fibrosis	90.9	2.3	6.8	0	0	100
Induration	93.2	2.3	4.5	0	0	100
Itching	97.7	0	2.3	0	0	100
Dry skin	97.7	2.3	0	0	0	100
Mastitis	95.5	0	4.5	0	0	100
Edema	97.7	0	2.3	0	0	100
Seroma	95.5	0	4.5	0	0	100
Erythema/hematoma	72.7	20.5	6.8	0	0	100
Epitheliolysis submammary	95.5	4.5	0	0	0	100
Radiation dermatitis	81.8	0	6.8	11.4	0	100

**Table 3:** Late toxicity > 90 days after IORT

Adverse event	Grade 0 (%)	Grade 1 (%)	Grade 2 (%)	Grade 3 (%)	Grade 4 (%)	*N* = 35 (%)
Hyperpigmentation	88.6	5.7	5.7	0	0	100
Skin pain	85.7	14.3	0	0	0	100
Fibrosis	88.6	11.4	0	0	0	100
Induration	97.2	2.8	0	0	0	100
Itching	88.6	8.6	2.8	0	0	100
Dry skin	94.3	5.7	0	0	0	100
Edema	97.1	0	2.9	0	0	100
Seroma	97.1	0	2.9	0	0	100
Erythema/hematoma	45.7	34.3	20	0	0	100
Epitheliolysis submammary	94.4	2.8	2.8	0	0	100
Epitheliolysis axillary	85.7	11.4	2.9	0	0	100
Radiation dermatitis	85.7	0	11.4	2.9	0	100

### Oncologic outcomes

**Fig. 1:**
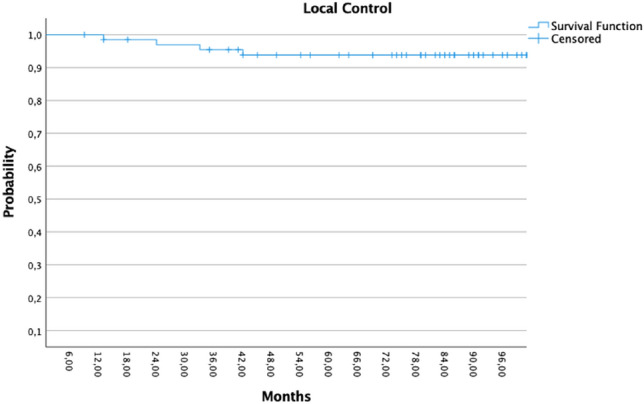
Kaplan–Meier showed the ipsilateral local control

**Fig. 2:**
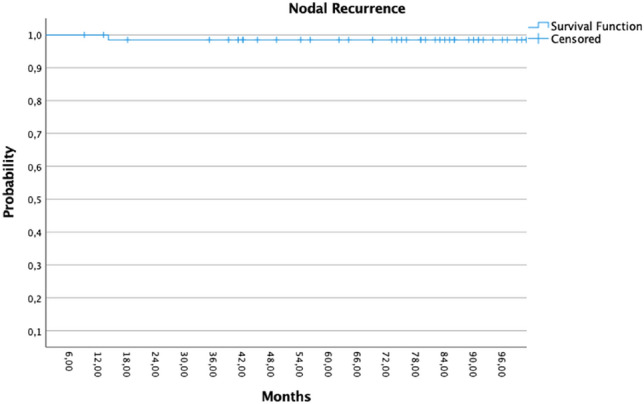
Kaplan–Meier showed the nodal control

The median duration of follow-up was 91.5 months (range 9–125 months). The local recurrence-free survival was 92.6% for all participants (5/68 recurrences). All patients with positive hormone receptors (*n* = 64) were administered adjuvant hormone treatment. Twenty patients received adjuvant chemotherapy, including six patients with lymph node metastases. There have been five recurrences in the tumor bed and ipsilateral breast with a median follow-up of 91 months (range 13–125 months). The 5-year local recurrence rate was 7% (95% CI 95.6–91.4%) and the contralateral breast recurrence rate (CBE) was 2.9% (95% CI 97.2–94.3%), Fig. 1 depicts the pertaining Kaplan–Meier plot. As can be seen in Fig. 2 nodal recurrences were rare events, only one patient had lymph node metastases after the last WBI.

**Fig. 3:**
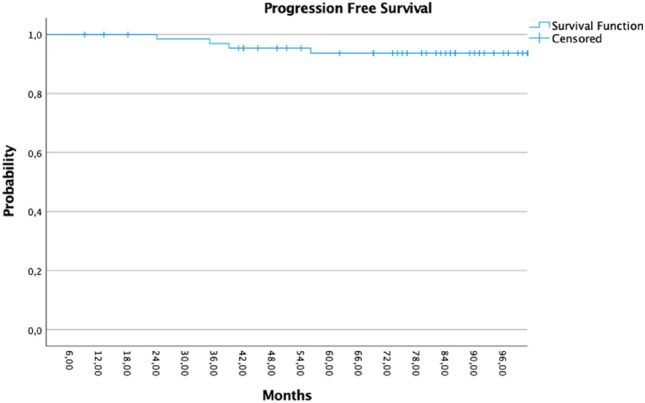
Kaplan–Meier showed the disease progression-free survival (PFS)

**Fig. 4:**
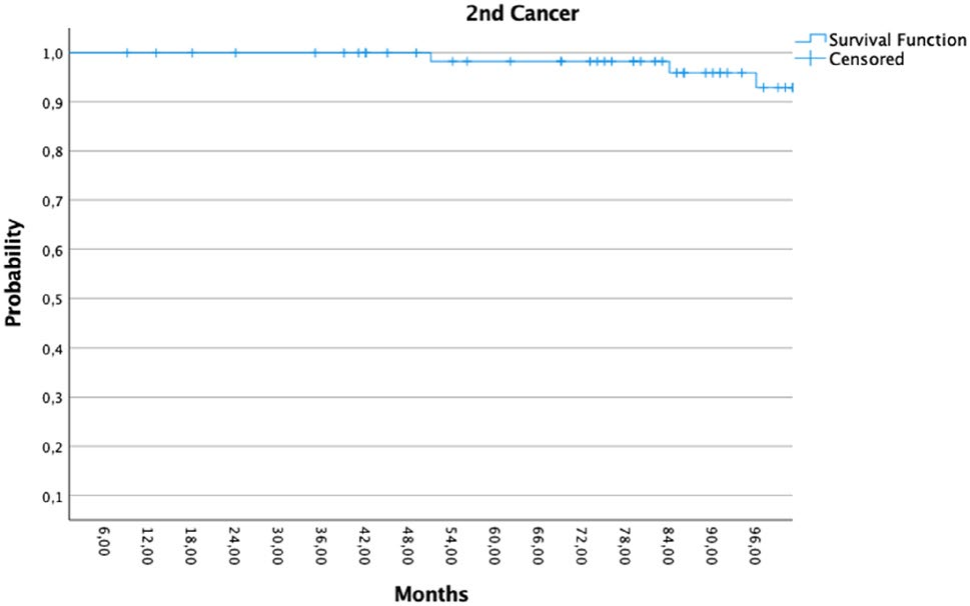
Kaplan–Meier showed the 2nd cancer progression-free survival (PFS)

**Fig. 5:**
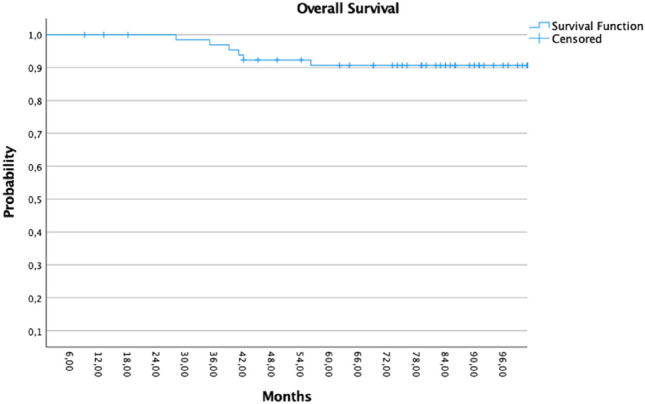
Kaplan–Meier showed the Overall Survival

Four patients developed distant metastases, and nine patients had passed away. Nine patients developed second cancer, 4/9 patients were with thoracal malignancies (two contralateral IDC and two bronchial cancer). Only one patient received adjuvant radiotherapy after first radiotherapy for local recurrence. The patient with local recurrence (pT4) received WBI with a 45 Gy with a 10 Gy boost over the lumpectomy area (Figs. 3, 4 and 5).

The risk of acute and chronic toxicity was not significantly associated with any specific factors, including applicator size, systemic treatment, or neoadjuvant chemotherapy, according to correlation analysis.

## Discussion

This single-institutional retrospective study presents preliminary findings on the feasibility, minimal toxicities, and relationship with suitable early oncological outcomes for breast cancer with IORT boost with 50-kV photons.

In the ELIOT Trial, electrons were employed to provide a single dose of 21 Gy to the tumor bed as opposed to WBRT with normofractionation (50 Gy in 25 fractions of 2 Gy, plus a 10 Gy tumor bed boost in 5 fractions) (Veronesi et al. 2013; Orecchia et al. 2021). The 5-, 10-, and 15-year LR rates for IORT-arm were 4.2%, 8.1%, and 12.6% with a median follow-up of 12.4 years compared to 0.5%, 1.1%, and 2.4% for patients treated with WBRT-arm (*p* 0.0001). Despite a fivefold difference in the local recurrence rate for IORT and a substantial difference in nodal recurrences (1.9% for IORT vs. 0.3% for WBRT, *p* = 0.01), there were no significant differences in the rates of distant metastasis or overall survival. The ELIOT Trial Group came to the conclusion, that IORT should be provided to patients with a low risk of local recurrence. ELIOT revealed a group with a particularly low risk as B1 cm, well-differentiated, luminal A tumors with a Ki67 < 14%. Notably, 5% of ELIOT IORT patients (those with C4-positive lymph nodes) underwent further WBRT. The ELIOT arm had a greater risk of recurrence than the WBI arm (11% vs. 2%, *p* 0.001), but there was no difference in overall survival between the two groups.

With similar methods, Wenz et al. (2010) showed a definite association between breast volume, applicator size, and degree of fibrosis. The same institution's long-term review of 400 patients after 78 months revealed minimal high-grade adverse events, primarily fibrosis and discomfort, and low rates of in-breast and axillary node recurrence. In the first three years following IORT and WBI, the bulk of late side effects manifested (Pez et al. 2020). Considering this, chronic toxicity may be underestimated given the little follow-up duration in our group, which had a median of only 28 months. Additionally, Wenz et al. showed that a possible shorter time gap (30 days) between IORT and WBI resulted in the development of higher-grade fibrosis grades II-III after a median of 36 months (Wenz et al. 2008). Early on, we included these findings and the advice to maintain a 5–6-week gap between IORT and WBI in our therapeutic practices. Only 4.2% (*n* = 9) of the patients in our sample had a gap of less than 30 days, and 12.7% (*n* = 27) of the patients had a total gap of less than 35 days, with a median gap of 54 days between IORT and WBI.

The TARGIT-A research, which was carried out in 11 nations (Vaidya et al. 2014), was one of the biggest studies that compared WBI with IORT. Women with unifocal ductal carcinomas who were older than 45 years old were included in this research. In patients who underwent IORT during a median follow-up of 2 years and 5 months, they found a 3.3% risk of local recurrence and a 3.9% death rate. Most of their tumors were grade 1 or 2 (85%), less than 2 cm (87%), ER positive (93%) and PR positive (82%) according to this research. There was no statistically significant difference between the IORT group (*n* = 1140) and the EBRT group (*n* = 1158) in terms of local recurrence-free survival (167 vs. 147 months, respectively; *p* = 0.28), mastectomy-free survival (170 vs. 175 months, respectively; *p* = 0.74), or mortality due to BC (65 vs. 57, respectively; *p* = 0.54) in a report with a longer follow Overall, they came to the conclusion that TARGIT-IORT was a potent option to EBRT for early-stage BC since it was non-inferior to EBRT during long-term follow-up.

A recent German study at the University of Freiburg Medical Center, Stoian et al. (2021) investigated the use of IORT to deliver a boost for the tumor bed after BCs in 214 patients with a median follow-up for the 28 (range 2–59) months. There were no grade 4 events, and the only acute grade 3 toxicities were 17 (8%) incidences of radiation dermatitis. The 3-year ipsilateral breast recurrence (IBRT) rate was 1.8% (95% CI 99.5–92.7%) while the contralateral breast recurrence (CBE) rate was 0.6% (95% CI 99.9–95.9%).

Another American study analyzed 1400 patients at a single institute (Silverstein et al. 2022). They also recruited patients for additional WBRT using a "risk-adapted" method similar to TARGIT A. Patients who violated any of the entrance risk criteria in this trial were instructed to get extra local therapy. However, 191 of 409 (47%) patients who failed to meet one or more of the criteria denied further local therapy. The 13 patients who refused further treatment had a 5-year likelihood of local recurrence of 8.0%, whereas the 167 patients with protocol violations who received supplemental WBRT had a rate of local recurrence of just 0.63%. This highlights the significance of include WBRT in IORT when encountering high-risk traits.

They investigated at a subset of 1175 patients who got IORT alone without re-excision, WBRT, or conversion to mastectomy. They had 60 local recurrences, with a 5-year chance of a local recurrence of 5.98%. This is a somewhat high rate of local recurrence; however, it is lower than in TARGIT B. The significant prevalence of local recurrence can be attributed to the fact that 190 of these patients failed one or more protocol criteria and hence should have gotten extra local therapy but did not. Silverstein et al. reported 5-year LR rate of 5.27% for the whole cohort. It raised to 5.98% for 1175 patients who had IORT as their sole source of local therapy.

Our report supports previous research utilizing IORT as a boost, showing a 5-year LR rate of about 7% and a comparable incidence of acute grade 3 and late grade 2 toxicities. This study's low rate of postoperative complications, which was quantitatively equivalent to earlier data, further supports the safety of IORT from this angle. Regarding long-term toxicity, our findings contrast well with no instance of grade III fibrosis (Table 3).

Despite these hopeful findings, this investigation's limitations must be addressed. The first one has to do with the study's single-institutional, retrospective design, meticulous patient selection for IORT, and consequently limited applicability to different patient groups. More thorough information on the restricted benefit/risk ratio for administering boost therapies to low-risk individuals has also been made available by newer data. The study's brief follow-up also restricts the insight it can provide into hazardous episodes and long-term recurrences/survival. In this sense, it is critical to collect longer-term outcomes from this and other cohorts. The capability of single-shot IORT to adequately cover enough target volumes with suitable dosages is a significant potential restriction.

## Conclusion

In conclusion, this study offers preliminary evidence that supports the use of IORT in the boost context. There were just a few higher-grade toxicities overall, a very low prevalence of postoperative problems, and favorable results for early breast cancer. The impending publication of randomized trials like TARGIT-B should support these findings.

## Data Availability

The datasets used and analyzed during the current study are available from the corresponding author on reasonable request.

## Abbreviations

LR: Local recurrence rate
IBTR: Ipsilateral in-breast tumor recurrence
CBE: Contralateral in-breast tumor recurrence
PFS: Progression-free survival
OS: Overall survival
IORT: Intraoperative radiation
WBI: Whole-breast irradiation
BCS: Breast-conserving surgery
RT: Radiotherapy
CTCAE: Common Terminology Criteria for Adverse Events
